# The Effects of Jiang-Zhi-Ning and Its Main Components on Cholesterol Metabolism

**DOI:** 10.1155/2012/928234

**Published:** 2012-05-09

**Authors:** Jianxin Chen, Huihui Zhao, Xueling Ma, Xiao Han, Liangtao Luo, Luya Wang, Jing Han, Bing Liu, Wei Wang

**Affiliations:** ^1^Beijing University of Chinese Medicine, 11 Bei San Huan Dong Lu, Chao Yang District, Beijing 100029, China; ^2^Institute of Chinese Materia Medica, China Academy of Chinese Medical Sciences, No. 16 Nanxiaojie, Dongzhimennei, Beijing 100700, China; ^3^Beijing Institute of Heart, Lung and Blood Vessel Diseases, Beijing Anzhen Hospital Affiliated to Capital Medical University, Beijing 100029, China

## Abstract

To examine how Jiang-Zhi-Ning (JZN) regulates cholesterol metabolism and compare the role of its four main components. We established a beagle model of hyperlipidemia, fed with JZN extract and collected JZN-containing serum 0, 1, 2, 4, and 6 h later. Human liver cells Bel-7402 were stimulated with 10% JZN-containing serum as well as the four main components of JZN and Atorvastatin. The mRNA expression of LDL receptor (LDL-R), 3-hydroxy-3-methyl-glutaryl-CoA reductase (HMG-CoAR), cytochrome P450 7A1 (CYP7A1), and acetyl-Coenzyme A acetyltransferase 2 (ACAT2) was measured by real-time PCR. LDL-R surface expression and LDL-binding and internalization were examined by flow cytometry. The results showed that JZN-containing serum significantly increased the mRNA expression of LDL-R, HMG-CoAR, and CYP7A1 in Bel-7402 cells. All the four components significantly increased the mRNA and protein expression of LDL-R and HMG-CoAR and decreased the mRNA and protein expression of ACAT2 in Bel-7402 cells. Hyperinand chrysophanol also markedly increased the mRNA expression of CYP7A1. Stimulation with stilbene glycosidesignificantly increased the surface expression of LDL-R and the binding and internalization of LDL. In conclusion, JZN and its four components have close relationship with the process of cholesterol metabolism, emphasizing their promising application as new drug candidates in the treatment of hyperlipidemia.

## 1. Introduction

Numerous studies have shown that cholesterol plays a key role in the development of atherosclerosis, which is the main pathological basis of cardiovascular diseases [1]. Increase in the level of serum total cholesterol, especially low-density lipoprotein (LDL) cholesterol, is of significant importance in developing diseases. Multiple clinical experiments have shown that lowering the level of serum total cholesterol, especially LDL cholesterol, can decrease lipid content in atherosclerotic plaques, lessen the shear force from blood on the cap of fibrous tissues, and reduce the secretion of proteases from foam cells that can hydrolyze extracellular matrix. This progress can stabilize plaques or even reduce their size, stop disease progression, and decrease cardiovascular morbidity and mortality [2].

Cholesterol metabolism is a complicated homeostasis involving multiple steps, including cholesterol absorption, synthesis, conversion, and modification. LDL receptor (LDL-R) plays a critical role in cholesterol absorption. In the serum, cholesterol mainly exists in the form of cholesterol ester and is carried and transported by lipoproteins, such as LDL, apolipoprotein B100 (Apo B100), and apolipoprotein E (Apo E). LDL-R binds to these lipoproteins and internalizes them into cells, providing lipids for cell proliferation and synthesis of steroid hormones and bile salt. These animals metabolized 7.1 pools of LDL-cholesterol (LDL-C) per day, and 79% of this degradation took place in the liver. Of this total turnover, the LDLR accounted for 88% while the remaining 12% was receptor independent. 91% of the receptor-dependent transport identified in these animals was located in the liver while only 38% of the receptor-independent uptake was found in this organ [3]. Indeed, a functional deficit in LDL-R is one of the major causes of hypercholesterolemia and atherosclerosis [4, 5].

Cholesterol in the body can be obtained from food intake or from liver biosynthesis, the latter accounting for generating 70% to 80% of serum total cholesterol [6]Thus, inhibiting cholesterol biosynthesis is an effective way to reduce total cholesterol levels. Cholesterol synthesis includes 30 steps of enzymatic reaction, where 3-hydroxy-3-methyl-glutaryl-CoA reductase (HMG-CoAR) is a rate-limiting enzyme. Currently available cholesterol-lowering drugs are mainly statins, whose main target is HMG-CoAR.

Another important step in cholesterol metabolism is the conversion of cholesterol into bile acid through cytochrome P450-meidiated oxidation. The rate-limiting enzyme for the dominant pathway of bile acid synthesis, the so-called classic pathway, is cytochrome P450 7A1 (CYP7A1). Human CYP7A1 gene defect can cause cholesterol accumulation in the liver, which has been associated with hypercholesterolemia [7]. In contrast, high-level expression of CYP7A1 increases the mRNA expression of LDL-R in liver cells and decreases the concentration of circulating LDL-cholesterol even in LDL-R-deficient mice [8]. In human, CYP7A1 expression is induced by its substrate cholesterol and inhibited by negative feedback from bile acid [9].

Another interesting participant in cholesterol metabolism is acetyl-CoA acetyltransferase 2 (ACAT2), which esterifies cholesterol in the small intestine and liver. Cholesterol esters synthesized by ACAT2 are packed into very-low-density lipoprotein and secreted into blood. It is observed in an atherosclerosis model of Apo E-deficient mice that the formation of atherosclerosis can be prevented by simultaneous knockout of ACAT2, suggesting that ACAT2-mediated cholesterol esterification is important for atherosclerosis [10].

Among all current lipid-regulating drugs, Atorvastatin shows the most significant clinical effects. Atorvastatin decreases the cholesterol level by inhibiting HMG-CoAR and promotes the transcription of LDL-R by activating the transcription factor steroid response element binding protein 2 (SREBP2). The defined targets and marked lipid-lowering effects of Atorvastatin have made it a major breakthrough in lipid-lowering drugs. Atorvastatin has now become a preferred first-line lipid-regulating drug. However, statins may cause serious side reactions, such as liver toxicity and statin myopathy, with symptoms including increases in alanine aminotransferase and aspartate aminotransferase, dermatomyositis and polymorphic myositis, and so forth. In severe cases, statins can lead to rhabdomyolysis. Therefore, it is imperative to develop novel, safe, and effective lipid-regulating drugs.

Traditional Chinese Medicine (TCM) has been used to prevent and cure atherosclerosis and lower lipid for thousands of years. Jiang-Zhi-Ning (JZN), a widely used ready-made Chinese medicine, is composed of stilbene glycoside (from ShouWu, fleeceflower root), hyperin (from ShanZha, fructus crataegi), nuciferine (from HeYe, folium nelumbinis), and chrysophanol (from JueMingZi, semen cassiae). JZN has been in clinical application for more than 1300 years and has been shown to significantly lower serum cholesterol levels. The four herbs in JZN, Fleeceflower Root, Fructus Crataegi, Folium Nelumbinis, and Semen Cassiae have been used in clinic on obesity for centuries as in “QianJinFang” (Prescriptions Worth Thousands Gold). Though at that time the disease is not called as hyperlipidemia, but recent research has confirmed that those herbs have significant effect on lowing serum cholesterol levels [11–15].

This study aims to examine the underlying mechanisms of the lipid-lowering role of JZN and compare how its four main components contribute to this function. Cholesterol metabolism after drug treatment was examined by analyzing the expression of LDL-R, HMG-CoAR, CYP7A1, and ACAT2.

## 2. Materials and Methods

### 2.1. Animals and Cell Lines

Adult laboratory beagles weighted between 10 ± 1 kg were provided by Beijing TongLi Laboratory Animal Culture Company (Beijing, China). Liver cell Bel-7402 was provided by Institute of Basic Medical Sciences of Chinese Academy of Medical Sciences (Beijing, China). After thaw, Bel-7402 cells were first cultured at 37°C in a CO_2_ incubator in RPMI-1640 culture solution containing 10% fetal bovine serum, 100 U/mL penicillin, and 100 *μ*g/mL streptomycin.

### 2.2. Preparation of JZN Extracts and the Four Components

Fleeceflower root is the processed product of the dried root of polygonum multiflorum thunb. Folium nelumbinis is dried leaf of nelumbo nucifera gaertn. Fructus crataegi and semen cassiae are dried mature fruits of *Crataegus pinnatifida* Bge. var. major N. E. Br. and Cassia obtusifolia L., respectively. All were purchased from Beijing TongRenTang Inc. (Beijing, China) and authenticated by Professor Guijun Zhang from Beijing University of Chinese Medicine.

Fleeceflower root (25 g) and folium nelumbinis (75 g) were mixed and 25-fold of 50% ethanol was added. The mixture was heated and refluxed for 1.5 h. Then, ethanol extract was swilled and the residues were filtered and distilled twice. The three extracts were combined and concentrated into ointment, which was named as whole solid I. Fructus crataegi (500 g) and semen cassiae (25 g) were mixed and 7-fold of water was added. The mixture was heated and refluxed for 2 h. Then, the liquid extract was swilled and the residues were filtered and distilled. The two liquid extracts were combined and concentrated into ointment, which was named as whole solid II. The two whole solids were mixed and then dried using decompression drying method at 50°C.

Hyperin (from ShanZha) and stilbene glycoside (from ShouWu) were dissolved in sterilized water. Nuciferine (from HeYe) was dissolved in hydrochloric acid and then the pH was adjusted to 7.0 by sodium hydroxide. Chrysophanol and Atorvastatin were dissolved in DMSO.

### 2.3. Preparation of JNZ-Containing Serum

To establish a beagle model of hyperlipidemia, beagles were adaptively fed for one week and then fed with high-fat feed for two months. The fat feed was composed of 88% of usual feed, 10% of lard, and 2% of cholesterol. Beagles with hyperlipidemia were then fasted for 12 h and fed with 0.4 g/kg of JZN extract. 10 mL of vein blood was withdrawn from the back limb of beagles after 0, 1, 2, 4, and 6 h. The blood was centrifuged at 3000 r/min for 10 min to obtain serums, which were heat-inactivated for 30 min at 56°C and filtered through 0.22 *μ*m micropore membrane. The concentration of serum LDL was measured using chemically selective inhibition method.

### 2.4. Isolation of LDL from Human Serum

The human serum was collected and centrifuged with gradient solution in a density of 1.0, 1.1, and 1.3 at 4500 r/min for 10 min. The yellow lipid in the center of the solution was extracted as LDL. After a 48-h dialysis in the dialysate, which contains 0.02 M Tris, 0.01% EDTA, and 0.9% NaCl, pH 7.4, the isolated LDL was kept at 4°C. The separation of LDL was confirmed by gelose gel electrophoresis with oil red O staining. LDL concentration was determined by the bicinchoninic acid method.

### 2.5. Stimulation of Bel-7402 Cells with JZN-Containing Serum

Bel-7402 cells were cultured in 6-well plates at 3∗10^5^ cells per mL in 10% 1640 culture media. Alternatively, after cells became 80% confluent, the 10% 1640 media were replaced by serum-free 1640 media. After starvation for 24 h to synchronize cells to the G_0_ phase, the serum-free media were replaced with 1 mL of 1640 culture media containing 10% JZN-containing serum from different time points. Isolated human LDL was added to the culture media to make the final LDL concentration in the media equal to the LDL concentrations in JZN-containing serum at different time points.

### 2.6. Stimulation of Bel-7402 Cells with Four Components of JZN

Bel-7402 cells in logarithmic growth were cultured in 35 mm or 100 mm flasks at 3∗10^5^ cells per mL and starved in serum-free 1640 culture media for 24 h. After cells became 80% confluent, they were treated with the four components of JZN or Atorvastatin. The effects of each component were examined at three different concentrations: the low, medium, and high levels. The three concentrations of stilbene glycoside, nuciferine, and chrysophanol were 1, 10, and 100 *μ*M. The three concentrations of hyperin were 0.1, 1, and 10 *μ*M, respectively. The concentration of Atorvastatin was 10 *μ*M. The drugs, after incubated in the CO_2_ incubator with 10% BSA for 2 h, were added to cells for 24 h.

### 2.7. RT-PCR

Total RNA was extracted from drug-stimulated Bel-7402 cells using Trizol one-step method and then reverse-transcribed into cDNA using AMV Reverse Transcriptase. RT-PCR was conducted by the CYBR green method using following primers (Beijing Bioko Biotechnology Company, Beijing, China). *β*-Actin: F: 5′-GGCATCCTCACCCTGAAGTA-3′; R: 5′-GGGGTGTTGAAGGTCTCAAA-3′. LDL-R: F: 5′-GCTTGTCTGTCACCTGCAAA-3′; R: 5′-AACTGCCGAGAGATGCACTT-3′. HMG-CoAR: F: 5′-CTGGGAGCATAGGAGGCTAC-3′; R: 5′-CCACCCACCGTTCCTATCTC-3′. CYP7A1: F: 5′-GTCTTTCCAGCCCTGGTAGC-3′; R: 5′-GAGGACCACGAGGTGTGTCT-3′. ACAT2: F: 5′-CAAGGAGGTGAAGGACAAGC-3′; R: 5′-ATTGGACATGCTCTCCATCC-3′. PCR reactions were as follows: one cycle of 5 min at 95°C; 35 cycles of 1 min at 94°C, 45 s at 60°C, and 1 min at 72°C; final extension of 10 min at 72°C.

### 2.8. Western Blot

Equal amount of protein (50 *μ*g) was loaded onto each lane for SDS-PAGE and transferred to membranes. Membranes were first stained with XXX primary antibodies, then with horseradish peroxidase-linked secondary antibodies, and developed with a ECL kit (Amersham, UK). Films were scanned and band intensity was quantified by ImageMaster Total Lab 1.0.

### 2.9. Flow Cytometry

Cells from the high-dosage group of stilbene glycoside treatment were washed twice with PBS and then once with serum-free medium. Cells were incubated for 1 h in serum-free medium containing 2% BSA and then washed with 0.5% BSA. For examining surface expression of LDL-R, cells were incubated with FITC-conjugated anti-LDL-R primary antibody and then analyzed by flow cytometry. For analyzing LDL binding and internalization, cells were incubated with 2 *μ*L DIL-LDL for 1 h at 4°C and 37°C, respectively, and then washed and analyzed by flow cytometry.

## 3. Results

### 3.1. Effects of JZN-Containing Serum on Cholesterol Metabolism in Liver Cells

We first established a beagle model of hyperlipidemia, fed these beagles with JZN extract, and collected their serum after 0, 1, 2, 4, and 6 h. In Bel-7402 cells, 10% of JZN-containing serum from these beagles significantly increased the mRNA expression of LDL-R, HMG-CoAR, and CYP7A1 after X h stimulation (*P* < 0.05, Table 1). The maximum effect was induced by 1-h JZN-containing serum for LDL-R and HMG-CoAR and by 2-h JZN-containing serum for CYP7A1 (Table 1). In contrast, 1-h JZN-containing serum significantly decreased the mRNA expression of ACAT2 (Table 1).

### 3.2. Effects of Four Components of JZN on Cholesterol Metabolism in Liver Cells (mRNA Analysis)

As a positive control, we first tested the effects of Atorvastatin on cholesterol metabolism. Atorvastatin treatment increased the mRNA expression of LDL-R, HMG-CoAR, and CYP7A1 and decreased the mRNA expression of ACAT2 (*P* < 0.05, Tables 2, 3, 4, and 5).

Stilbene glycoside and nuciferine showed a similar pattern of influence on cholesterol metabolism in liver cells. Compared with the no-drug control group, mRNA expression of LDL-R and HMG-CoAR in liver cells was significantly increased after stilbene glycoside or nuciferine stimulation (*P* < 0.05, Tables 2 and 3). Stimulation with stilbene glycoside (median and high doses) or nuciferine (high dose) significantly decreased mRNA expression of ACAT2 (*P* < 0.01, Tables 2 and 3). CYP7A1 mRNA expression was not affected by stilbene glycoside or nuciferine (*P* > 0.05, Tables 2 and 3).

Hyperin and chrysophanol showed similar influence on cholesterol metabolism in liver cells. Compared with the control group, stimulation with hyperin or chrysophanol also significantly increased the mRNA expression of LDL-R and HMG-CoAR (*P* < 0.05, Tables 4 and 5). Different from stilbene glycoside and nuciferine, hyperin (low and medium doses) and chrysophanol (medium and high doses) significantly increased Cyp7A1 mRNA levels (*P* < 0.05, Tables 4 and 5). Expression of ACAT2 mRNA was significantly reduced by hyperin (medium and high doses) or chrysophanol (all three doses) treatment (*P* < 0.01, Tables 4 and 5).

### 3.3. Effects of Four Components of JZN on Cholesterol Metabolism in Liver Cells (Protein Analysis)

**Figure 1:**
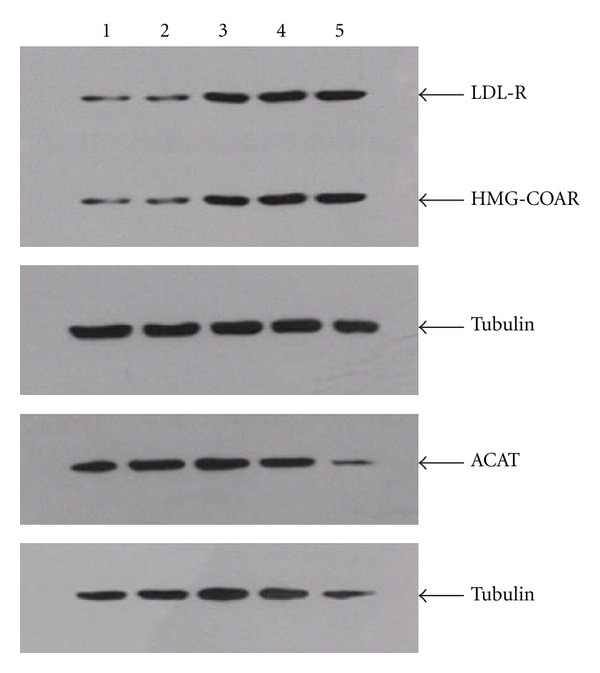
LDL-R, HMG-CoAR, and ACAT2 protein expression under stilbene glycoside treatment. Notes: Lane 1: no-drug control; 2: low dose of stilbene glycoside; 3: medium dose of stilbene glycoside; 4: high dose of stilbene glycoside; 5: Atorvastatin.

**Figure 2:**
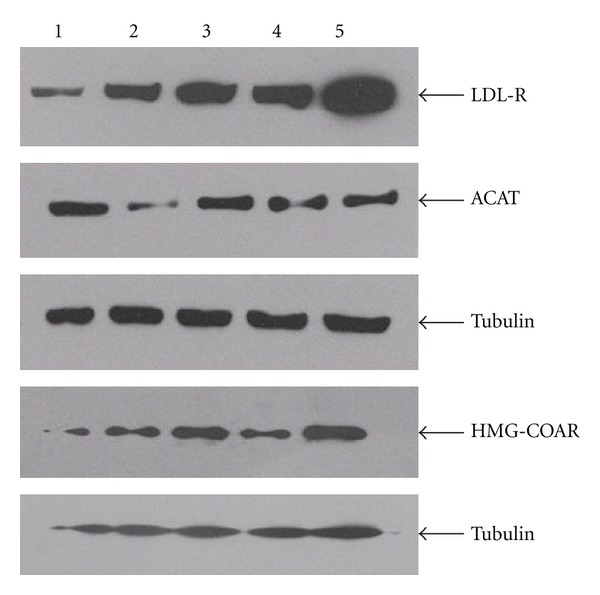
LDL-R, HMG-CoAR, and ACAT2 protein expression under nuciferine treatment. Notes: Lane 1: no-drug control; 2: low dose of nuciferine; 3: medium dose of nuciferine; 4: high dose of nuciferine; 5: Atorvastatin.

**Figure 3:**
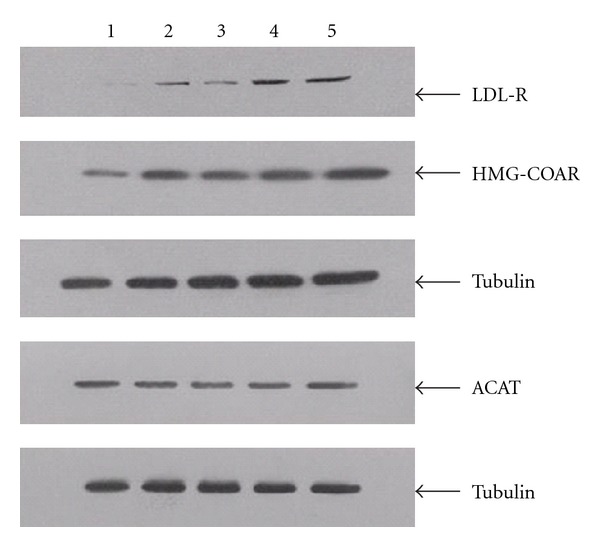
LDL-R, HMG-CoAR, and ACAT2 protein expression under hyperin treatment. Notes: Lane 1: no-drug control; 2: low dose of hyperin; 3: medium dose of hyperin; 4: high dose of hyperin; 5: Atorvastatin.

**Figure 4:**
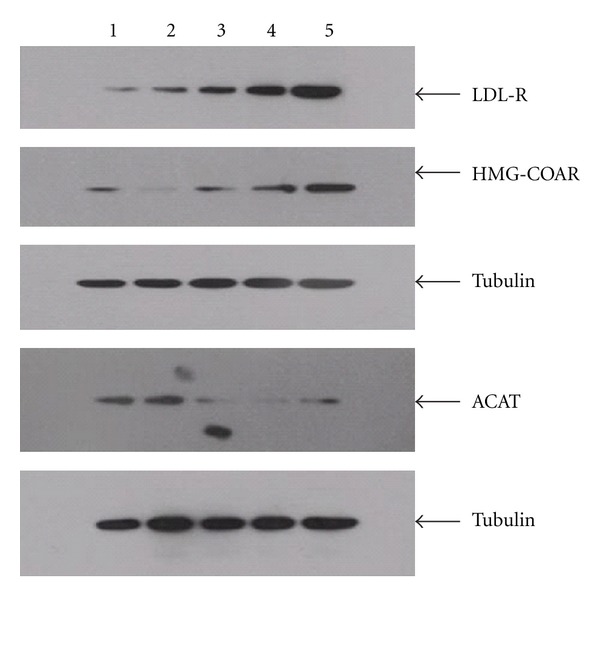
LDL-R, HMG-CoAR, and ACAT2 protein expression under chrysophanol treatment. Notes: Lane 1: No-drug control; 2: Low dose of chrysophanol; 3: Medium dose of chrysophanol; 4: High dose of chrysophanol; 5: Atorvastatin.

Atorvastatin and the four components of JZN showed similar affects. Protein expression of LDL-R and HMG-CoAR was significantly increased under all treatments (*P* < 0.01, Tables 6, 7, 8, and 9, Figures 1, 2, 3, and 4). In contrast, ACAT2 protein expression was significantly reduced under all treatments (*P* < 0.01 except for low dose of stilbene glycoside, Tables 6, 7, 8, and 9, Figures 1, 2, 3, and 4).

### 3.4. Effects of Stilbene Glycoside Treatment on Surface Level of LDL-R

**Figure 5:**
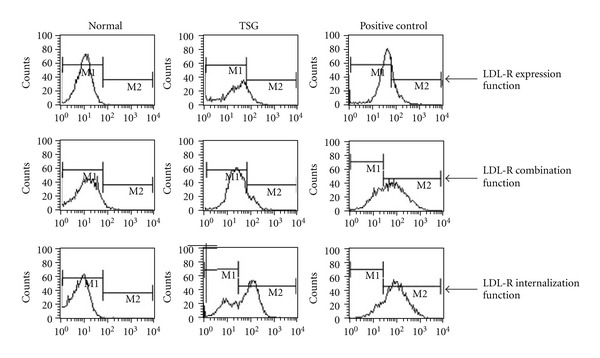
The effects of stilbene glycoside treatment on the surface expression of LDL-R. Treatment of stilbene glycoside or atorvastatin increased LDL-R surface expression as well as LDL binding and internalization.

Our flow cytometry analysis showed that similar to Atorvastatin, stilbene glycoside significantly increased surface expression of LDL-R, as well as binding and internalization of LDL (*P* < 0.01, Table 10 and Figure 5).

## 4. Discussion

Hyperlipidemia causes progressive atherosclerosis, which is the major cause of cardiovascular diseases. Clinically, 80% to 90% of acute coronary events are triggered by sudden collapse of atherosclerotic plaques [16]. The stability of plaques is related to lipid content: the higher the lipid content, the lower the stability. In addition, hyperlipidemia is also an important risk factor for hypertension, impaired glucose tolerance, and diabetes. Hyperlipidemia can also lead to hepatic adipose infiltration, cirrhosis, gallstones, pancreatitis, fundus hemorrhage, blindness, peripheral vascular disease, claudication, and hyperuricemia. Some primary and familial hyperlipidemia may also cause tendon-like, nodular, palm print, periorbital xanthomas, and arcus juvenilis. Therefore, hyperlipidemia treatment has become a hot topic in recent years.

Many different types of lipid-regulating drugs, including resins, statins, niacin, and unsaturated fatty acid lipid-regulating drugs, have been developed. However, most of these drugs only focus on one step of cholesterol metabolism. Few currently available drugs target cholesterol synthesis, absorption, modification, and conversion all together. In addition, most lipid-regulating drugs cause serious side effects, including gastrointestinal problems and liver and kidney dysfunction.

Numerous lipid-lowering formulae have been recorded in TCM. However, their application has been limited due to a lack of thorough understanding of both their effective ingredients and underlying mechanisms. There is also no standardized quality control in drug preparation. Therefore, it is imperative to examine active ingredients from known lipid-lowering TCM for their pharmacological effects. This has been proven to be an effective strategy to develop novel, more efficacious lipid-regulating drugs with fewer side effects. It has been reported that the activity of LDL-R can be enhanced by tea polyphenol as well as the water-soluble and ethanol-soluble extracts of turmeric [17, 18]. The expression of LDL-R in rat liver is increased by guanxinkang decoction that is composed of astragalus, trichosanthes, scalion white, and salvia miltiorrhiza [19, 20]. The expression of LDL-R and high-density lipoprotein receptor is also increased by tiaogandaozhuo decoction consisting of processed polygonum multiflorum, radix bupleuri, cassia, alisma, salvia miltiorrhiza, fructus leonuri, tumeric, cattail pollen, and so forth [21]. Up to now, studies in this area have mainly focused on regulation on LDL. The current study aims to extend research on lipid-lowering TCM to cholesterol absorption, synthesis, conversion, and other steps in cholesterol metabolism.

JZN shows significant cholesterol-lowering effects. Polygonum multiflorum thunb, hawthorn, lotus leaf, and cassia seed are all frequently used as lipid-lowering TCM. Total glycoside from polygonum multiflorum thunb shows significant lipid-lowering and antioxidant effects [22]. The total cholesterol and triglyceride values in hyperlipidemic mice are significantly reduced by hawthorn flavonoids of different purity [23]. Furthermore, different proportions of cassia seed extract and hawthorn extract have been shown to reduce the levels of total cholesterol, triglyceride, LDL-cholesterol, and Apo B in hyperlipidemic mice [24].

However, the current understanding of JZN or its four major components is still far from satisfactory. Stilbene glycoside has been shown to exert neuroprotectivity, antioxidation, and lipid-lowering effects [25, 26]. But its mechanisms remain unclear. Pharmacological studies of hyperoside mainly focus on analgesia and the protection of cardiovascular system whereas its effect on hyperlipidemia has not been reported [27]. The role of nuciferine has been studied in models of obese hyperlipidemic rats [28]. The function of chrysophanol is hardly known.

In the current study, we first examined the role of JZN in lipid metabolism in liver cells. It is worth noting that for a long time, a routine TCM pharmacological experiment is conducted as follows: ingredients of TCM are first extracted and separated and then added directly to an *in vitro* system (e.g., cell culture), and the effects of various ingredients are added to illustrate the effectiveness of a TCM recipe. Research on pharmacodynamic material basis and mechanism of Chinese herbal compound is still the difficult and hot spot in Chinese medicine research. Study on serum pharmacochemistry of herbal compound directly from the components that absorbed into the body directly narrows the scope of constituent research and provides a new way for herbal compound research. But it is still at the exploratory stage, there are many difficulties in practical research. In our experiment, we found that the serum medicine concentration is low in case of Chinese herbal compound taken by oral and due to the experimental conditions and technical limitations, which is still a challenge in drug-testing. However, this method neglects the following characteristics of TCM: (1) in most cases, TCM is taken orally and thus the ingredients entering blood after metabolism in the gastrointestinal tract and liver may differ significantly from the original ingredients; (2) the components of TCM may interact and cross-talk and therefore, the overall effect of TCM does not equal the sum of the effects of individual ingredients; (3) the physical and chemical properties of TCM components in extracts are not constant from study to study, making it difficult to reach consensus conclusions. Based on the above considerations, Shinichi Tashiro proposed using medicine-containing serum for *in vitro *pharmacological studies [29]. Chinese herbal compound exerts its pharmacodynamics effect after oral administration and gastrointestinal absorption into blood and body interactions. Applying serum pharmacology method, the use of the medicine-containing serum *in vitro* experiment is more closely related to the real process of exerting pharmacological effects *in vivo* environment. This method provides a new way for pharmacodynamic material basis research of Chinese herbal compound.

Medicine-containing serum is collected from animals fed with TCM after a certain period of time. This TCM serum method mimics the genuine route of TCM to take effects *in vivo* and thus provides more information about the pharmacological role of TCM. We adopted this method and demonstrated that in liver Bel-7402 cells, 10% JZN-containing serum significantly increases the mRNA expression of LDL-R, HMG-CoAR, and CYP7A1. Since it is crucial to observe the efficacy of different time points of JZN-containing serum to further determine which point in time to extract and separate the serum-containing material. According to the reported drug metabolism *in vivo*, about 80 percent reported that the process of drug metabolism is within two hours. So our experiments set 0, 1, 2, 4, 6 hours after administration as five time points. However, due to the complexity of endogenous components of the serum itself, if we use blank serum as a control, it will be another difficulty in determining the time points of blank serum. In this study, we use the LDL concentration in the medium calibration to ensure a consistent amount of stimulation.The maximum induction effect is achieved by 1-h JZN-containing serum for the former two molecules and by 2-h JZN-containing serum for the last one. JZN-containing serum collected after 1 h significantly reduces the mRNA expression of ACAT2. The different effects at different time points may reflect changes in effective ingredients of the plasma drug following drug uptake.

Cholesterol absorption and synthesis are negatively regulated by their end products. When cholesterol levels are low (e.g., when treated with Atorvastatin), the transcription factor SREBP is activated and triggers the transcription of LDL-R and HMG-CoAR [30]. The dominant regulator of CYP7A1 transcription is liver X receptor *α* (LXR*α*), a sterol-dependent nuclear receptor [31, 32]. The LXR response element DR4 has been found to interact with the promoter region of CYP7A1 in human and mice [33]. The current study suggested that JZN-containing serum increases the mRNA levels of LDL-R, HMG-CoAR, and CYP7A1 through the SREBP and LXR transcription pathways.

In this study, all four major components of JZN, stilbene glycoside, nuciferine, hyperin, and chrysophanol, significantly increase the mRNA and protein expression of LDL-R and HMG-CoAR and significantly reduce the mRNA and protein expression of ACAT2. Hyperin and chrysophanol also significantly increase the mRNA expression of CYP7A1. In addition, stilbene glycoside increases the surface expression of LDL-R as well as the surface binding and internalization of LDL. Our results showed that the components of JZN may exert lipid-lowering effects by increasing the surface expression and activity of LDL-R in liver cells, inhibiting intracellular cholesterol synthesis, increasing the conversion of cholesterol into bile acid, and reducing ACAT2-mediated cholesterol esterification. Our study suggested that these four components of JZN are promising candidates for developing new medications to treat hyperlipidemia and related diseases.

## Figures and Tables

**Table 1:** The effects of JZN-containing serum on cholesterol metabolism in liver cells.

Time point	LDL-R	HMG-CoAR	CYP7A1	ACAT2
0 h	1	1	1	1
1 h	3.66 ± 0.07**	1.95 ± 0.04*	1.74 ± 0.03**	0.73 ± 0.07
2 h	1.48 ± 0.03*	0.97 ± 0.11	1.98 ± 0.06**	0.87 ± 0.21
4 h	1.11 ± 0.08	1.27 ± 0.06*	1.26 ± 0.08*	1.2 ± 0.04
6 h	0.87 ± 0.12	0.94 ± 0.09	0.73 ± 0.07	0.99 ± 0.09

Note: comparison with 0-h time point, *P < 0.05, **P < 0.01.

**Table 2:** The effects of stilbene glycoside treatment on cholesterol metabolism in liver cells (mRNA analysis).

Treatment	LDL-R	HMG-CoAR	CYP7A1	ACAT2
No stilbene glycoside	0.65 ± 0.60	0.70 ± 0.05	0.24 ± 0.05	0.98 ± 0.06
Stilbene glycoside: low dose	0.74 ± 0.04*	0.90 ± 0.08**	0.21 ± 0.07	1.07 ± 0.07
Stilbene glycoside: medium dose	0.99 ± 0.12**	0.87 ± 0.12**	0.19 ± 0.05	0.85 ± 0.22**
Stilbene glycoside: high dose	1.26 ± 0.36**	1.02 ± 0.19**	0.26 ± 0.09	0.87 ± 0.32**
Atorvastatin	1.15 ± 0.14**	1.03 ± 0.24**	0.53 ± 0.04	0.65 ± 0.08*

Note: comparison with no-drug control group, *P < 0.05, **P < 0.01.

**Table 3:** The effects of nuciferine treatment on cholesterol metabolism in liver cells (mRNA analysis).

Treatment	LDL-R	HMG-CoAR	CYP7A1	ACAT2
No nuciferine	1	1	1	1
Nuciferine: low dose	1.86 ± 0.84**	2.18 ± 0.26**	0.94 ± 0.17	0.97 ± 0.24
Nuciferine: medium dose	2.95 ± 0.25**	2.58 ± 0.14**	0.93 ± 0.26	0.95 ± 0.28
Nuciferine: high dose	3.57 ± 1.01**	2.96 ± 0.25**	1.15 ± 0.31	0.78 ± 0.19**
Atorvastatin	5.83 ± 0.19**	6.58 ± 0.28**	1.29 ± 0.12*	0.49 ± 0.33**

Note: comparison with no-drug control group, *P < 0.05, **P < 0.01.

**Table 4:** The effects of hyperin on cholesterol metabolism in liver cells (mRNA analysis).

Treatment	LDL-R	HMG-CoAR	CYP7A1	ACAT2
No hyperin	1	1	1	1
Hyperin: low dose	1.64 ± 0.27*	2.18 ± 0.19**	1.21 ± 0.18*	1.03 ± 0.21
Hyperin: medium dose	2.82 ± 0.36**	3.29 ± 0.31**	1.57 ± 0.22**	0.65 ± 0.36**
Hyperin: high dose	2.66 ± 0.24**	2.87 ± 0.25**	1.06 ± 0.19	0.61 ± 0.29**
Atorvastatin	5.82 ± 0.27**	6.48 ± 0.29**	1.29 ± 0.45**	0.49 ± 0.38**

Note: comparison with no-drug control group, *P < 0.05, **P < 0.01.

**Table 5:** The effects of chrysophanol treatment on cholesterol metabolism in liver cells (mRNA analysis).

Treatment	LDL-R	HMG-CoAR	CYP7A1	ACAT2
No chrysophanol	0.42 ± 0.07	0.41 ± 0.11	0.28 ± 0.07	0.64 ± 0.15
Chrysophanol: low dose	0.62 ± 0.04*	0.54 ± 0.19*	0.31 ± 0.12	0.42 ± 0.16**
Chrysophanol: medium dose	0.75 ± 0.03**	0.72 ± 0.14**	0.33 ± 0.09*	0.37 ± 0.18**
Chrysophanol: high dose	0.88 ± 0.09**	0.81 ± 0.22**	0.37 ± 0.14**	0.30 ± 0.07**
Atorvastatin	0.93 ± 0.07**	0.86 ± 0.18**	0.49 ± 0.13*	0.31 ± 0.13**

Note: comparison with no-drug control group, *P < 0.05, **P < 0.01.

**Table 6:** The effects of stilbene glycoside treatment on cholesterol metabolism in liver cells (protein analysis).

Treatment	LDL-R	HMG-CoAR	ACAT2
No stilbene glycoside	0.32 ± 0.02	0.38 ± 0.05	1.24 ± 0.04
Stilbene glycoside: low dose	0.41 ± 0.06**	0.44 ± 0.07	1.26 ± 0.23
Stilbene glycoside: medium dose	1.09 ± 0.05**	0.96 ± 0.21**	1.07 ± 0.25
Stilbene glycoside: high dose	1.27 ± 0.07**	1.20 ± 0.24**	1.02 ± 0.31
Atorvastatin	1.58 ± 0.03**	1.77 ± 0.08**	0.51 ± 0.06**

Note: comparison with no-drug control group, *P < 0.05, **P < 0.01.

**Table 7:** The effects of nuciferine treatment on cholesterol metabolism in liver cells (protein analysis).

Treatment	LDL-R	HMG-CoAR	ACAT2
No nuciferine	0.25 ± 0.02	0.26 ± 0.03	1.23 ± 0.02
Nuciferine: low dose	0.60 ± 0.11**	0.48 ± 0.16**	0.76 ± 0.16**
Nuciferine: medium dose	0.91 ± 0.14**	0.53 ± 0.14**	0.87 ± 0.25**
Nuciferine: high dose	0.98 ± 0.26**	0.51 ± 0.23*	0.71 ± 0.18**
Atorvastatin	1.68 ± 0.21**	0.68 ± 0.19**	0.58 ± 0.22**

Note: comparison with no-drug control group, *P < 0.05, **P < 0.01.

**Table 8:** The effects of hyperin on cholesterol metabolism in liver cells (protein analysis).

Treatment	LDL-R	HMG-CoAR	ACAT2
No hyperin	0.17 ± 0.05	0.73 ± 0.03	0.74 ± 0.06
Hyperin: low dose	0.33 ± 0.08**	1.41 ± 0.07**	0.53 ± 0.02**
Hyperin: medium dose	0.29 ± 0.11*	1.47 ± 0.16**	0.48 ± 0.15**
Hyperin: high dose	0.62 ± 0.09**	1.54 ± 0.07**	0.57 ± 0.16*
Atorvastatin	0.86 ± 0.13**	2.69 ± 0.25**	0.52 ± 0.18*

Note: comparison with no-drug control group, *P < 0.05, **P < 0.01.

**Table 9:** The effects of chrysophanol treatment on cholesterol metabolism in liver cells (protein analysis).

Treatment	LDL-R	HMG-CoAR	ACAT2
No chrysophanol	0.23 ± 0.08	0.25 ± 0.04	0.36 ± 0.12
Chrysophanol: low dose	0.38 ± 0.11*	0.27 ± 0.17	0.32 ± 0.06
Chrysophanol: medium dose	0.61 ± 0.15**	0.29 ± 0.12	0.21 ± 0.11*
Chrysophanol: high dose	0.95 ± 0.13**	0.51 ± 0.14**	0.19 ± 0.09*
Atorvastatin	1.61 ± 0.26**	0.97 ± 0.28**	0.22 ± 0.07*

Note: comparison with no-drug control group, *P < 0.05, **P < 0.01.

**Table 10:** The effects of stilbene glycoside treatment on the surface expression of LDL-R.

Treatment	LDL-R Expression	LDL Binding	LDL Internalization
No-drug	11.68 ± 3.72	18.47 ± 2.34	7.94 ± 2.95
Stilbene glycoside	24.42 ± 5.21**	34.06 ± 6.29**	81.01 ± 5.22**
Atorvastatin	51.07 ± 6.34**	109.88 ± 10.60**	135.23 ± 17.63**

Note: comparison with no-drug control group, **P < 0.01.

## References

[1] Blankstein R, Budoff MJ, Shaw LJ, et al (2011). Predictors of coronary heart disease events among asymptomatic persons with low low-density lipoprotein cholesterol MESA (Multi-Ethnic Study of Atherosclerosis). *Journal of the American College of Cardiology*.

[2] Gotto AM (1995). Lipid lowering, regression, and coronary events: a review of the interdisciplinary council on lipids and cardiovascular risk intervention, seventh council meeting. *Circulation*.

[3] Osono Y, Woollett LA, Herz J, Dietschy JM (1995). Role of the low density lipoprotein receptor in the flux of cholesterol through the plasma and across the tissues of the mouse. *Journal of Clinical Investigation*.

[4] Kobayashi T, Ito T, Shiomi M (2011). Roles of the WHHL rabbit in translational research on hypercholesterolemia and cardiovascular diseases. *Journal of Biomedicine and Biotechnology*.

[5] Huijgen R, Vissers MN, Kindt I (2011). Assessment of carotid atherosclerosis in normocholesterolemic individuals with proven mutations in the LDL-receptor or apolipoprotein B genes. *Circulation: Cardiovascular Genetics*.

[6] Kawasaki M, Miura Y, Funabiki R (2010). Comparison of the effects on lipid metabolism of dietary methionine and cystine between hepatoma-bearing and normal rats. *Bioscience, Biotechnology, and Biochemistry*.

[7] Pullinger CR, Eng C, Salen G (2002). Human cholesterol 7*α*-hydroxylase (CYP7A1) deficiency has a hypercholesterolemic phenotype. *Journal of Clinical Investigation*.

[8] Ratliff EP, Gutierrez A, Davis RA (2006). Transgenic expression of CYP7A1 in ldl receptor-deficient mice blocks diet-induced hypercholesterolemia. *Journal of Lipid Research*.

[9] Björkhem I, Araya Z, Rudling M, Angelin B, Einarsson C, Wikvall K (2002). Differences in the regulation of the classical and the alternative pathway for bile acid synthesis in human liver. no coordinate regulation of CYP7A1 and CYP27A1. *Journal of Biological Chemistry*.

[10] Wilcox LJ, Borradaile NM, De Dreu LE, Huff MW (2001). Secretion of hepatocyte apob is inhibited by the flavonoids, naringenin and hesperetin, via reduced activity and expression of ACAT2 and MTP. *Journal of Lipid Research*.

[11] Guan JH, Xue Z, Ren JB (2001). Experimental research on the fat-rculating and anti-atheroscleorsis effect of JiangZhiNing. *Pharmacology and Clinics of Chinese Materia Medica*.

[12] Han X, Wu CA, Wang W (2008). Mechanism research of stilbene glucoside from polygonum multiflovum thunb on hyperlipemia. *Chinese Archives of Traditional Chinese Medicine*.

[13] Huang D, Shi YG (2005). Interventional effects of hawthorn, konjac and their compound on the levels of lipid and NO in plasma of rats being induced to hyperlipidemia. *Acta Academiae Medicinae Militaris Tertiae*.

[14] Zhang R, Feng ML, Wu YP (2005). Experimental study on the active situs of fetid cassia seed to reduce blood lipid and their dose-effect relation. *Chinese Remedies & Clinics*.

[15] Yang XY, Zhou YP, Dai LG (2005). Antilipemic effect of extracts of ampelopis grossedentata and lotus leaf (EAGLL) on rats with hypercholesterolemia. *Chinese Journal of Laboratory Animal Science*.

[16] Peeters W, Moll FL, Vink A (2011). Collagenase matrix metalloproteinase-8 expressed in atherosclerotic carotid plaques is associated with systemic cardiovascular outcome. *European Heart Journal*.

[17] Bursill CA, Roach PD (2006). Modulation of cholesterol metabolism by the green tea polyphenol (-)-epigallocatechin gallate in cultured human liver (HepG2) cells. *Journal of Agricultural and Food Chemistry*.

[18] Zhang WL, Liu DW, Wo XD, Zhang YH, Jin MM, Ding ZS (1999). Effects of curcuma longa on proliferation of cultured bovine smooth muscle cells and on expression of low density lipoprotein receptor in cells. *Chinese Medical Journal*.

[19] Li J, Yu L, Li N, Wang H (2000). Astragalus mongholicus and angelica sinensis compound alleviates nephrotic hyperlipidemia in rats. *Chinese Medical Journal*.

[20] Mao MJ, Hu JP, Chen FR, Zhang YY, Liu P (2011). Effects of chinese herbal medicine guanxinkang on lipid metabolism and serum c-reactive protein, amyloid a protein and fibrinogen in apolipoprotein e-knockout mice with atherosclerosis. *Journal of Chinese Integrative Medicine*.

[21] Wang Xl, Zhang HX, Lu YZ, Fan YC (2002). Effects of Tiaogandaozhuo Decoction on the Liver’s lipid content of rabbit with experimental atherosclerosis and the Lipoprotein Receptor in the Hepatocyte Membrane Vultured in Vitro. *Chinese Journal of Traditional Medical Science and Technology*.

[22] Yang PY, Almofti MR, Lu L (2005). Reduction of atherosclerosis in cholesterol-fed rabbits and decrease of expressions of intracellular adhesion molecule-1 and vascular endothelial growth factor in foam cells by a water-soluble fraction of polygonum multiflorum. *Journal of Pharmacological Sciences*.

[23] Xie W, Sun C, Liu S (2009). Effect of hawthorn flavanone on blood-fat and expression of lipogenesis and lipolysis genes of hyperlipoidemia model mouse. *Zhongguo Zhongyao Zazhi*.

[24] Li HB, Fang KY, Lü CT, Li XE (2007). Study on lipid-regulating function for the extracts and their prescriptions from semen cassiae and fructus crataegi. *Zhong Yao Cai*.

[25] Ryu G, Ju JH, Park VJ (2002). The radical scavenging effects of stilbene glycosides from poygonum multiflorum. *Archives of Pharmacal Research*.

[26] Lv L, Gu X, Tang J, Ho CT (2007). Antioxidant activity of stilbene glycoside from Polygonum multiflorum Thunb in vivo. *Food Chemistry*.

[27] Da Silva KL, Dos Santos ARS, Mattos PEO, Yunes RA, Delle-Monache F, Cechinel-Filho V (2001). Chemical composition and analgesic activity of calophyllum brasiliense leaves. *Therapie*.

[28] Lu YL, He YQ, Wang M (2010). Characterization of nuciferine metabolism by p450 enzymes and uridine diphosphate glucuronosyltransferases in liver microsomes from humans and animals. *Acta Pharmacologica Sinica*.

[29] Wu Z, Wu LJ, Tashiro S, Onodera S, Ikejima T (2005). Phosphorylated extracellular signal-regulated kinase up-regulated p53 expression in shikonin-induced hela cell apoptosis. *Chinese Medical Journal*.

[30] Tremblay AJ, Lamarche B, Lemelin V (2011). Atorvastatin increases intestinal expression of npc1l1 in hyperlipidemic men. *Journal of Lipid Research*.

[31] Chiang JYL, Kimmel R, Stroup D (2001). Regulation of cholesterol 7*α*-hydroxylase gene (CYP7A1) transcription by the liver orphan receptor (lxr*α*). *Gene*.

[32] Gupta S, Pandak WM, Hylemon PB (2002). LXR alpha is the dominant regulator of CYP7A1 transcription. *Biochemical and Biophysical Research Communications*.

[33] Luo Y, Tall AR (2000). Sterol upregulation of human cetp expression in vitro and in transgenic mice by an lxr element. *Journal of Clinical Investigation*.

